# Direct phone communication to primary care physician to plan discharge from hospital: feasibility and benefits

**DOI:** 10.1186/s12913-021-07398-w

**Published:** 2021-12-18

**Authors:** Lukas Enzinger, Perrine Dumanoir, Bastien Boussat, Pascal Couturier, Patrice Francois

**Affiliations:** 1 Service Universitaire de Gériatrie et Gérontologie Clinique, Centre Hospitalier Universitaire Grenoble Alpes, Grenoble, France; 2 Centre Gérontologique Sud, CHU Grenoble-Alpes, avenue de Kimberley, CS 90338, 38434 Echirolles, Cedex France; 3 Département Universitaire de Médecine Interne, CHU Grenoble Alpes, Grenoble, France; 4 Service d’épidémiologie et évaluation médicale, CHU Grenoble Alpes, Grenoble, France; 5 Laboratoire TIMC-IMAG, UMR 5525 CNRS, Univ. Grenoble Alpes, Grenoble, France

**Keywords:** Patient discharge, Communication, Primary care physicians, Hospitalists, Interprofessional relations, Telephone

## Abstract

**Background:**

The discharge summary is the main vector of communication at the time of hospital discharge, but it is known to be insufficient. Direct phone contact between hospitalist and primary care physician (PCP) at discharge could ensure rapid transmission of information, improve patient safety and promote interprofessional collaboration. The objective of this study was to evaluate the feasibility and benefit of a phone call from hospitalist to PCP to plan discharge.

**Methods:**

This study was a prospective, single-center, cross-sectional observational study. It took place in an acute medicine unit of a French university hospital. The hospitalist had to contact the PCP by telephone within 72 h prior discharge, making a maximum of 3 call attempts. The primary endpoint was the proportion of patients whose primary care physician could be reached by telephone at the time of discharge. The other criteria were the physicians’ opinions on the benefits of this contact and its effect on readmission rates.

**Results:**

275 patients were eligible. 8 hospitalists and 130 PCPs gave their opinion. Calls attempts were made for 71% of eligible patients. Call attempts resulted in successful contact with the PCP 157 times, representing 80% of call attempts and 57% of eligible patients. The average call completion rate was 47%. The telephone contact was perceived by hospitalist as useful and providing security. The PCPs were satisfied and wanted this intervention to become systematic. Telephone contact did not reduce the readmission rate.

**Conclusions:**

Despite the implementation of a standardized process, the feasibility of the intervention was modest. The main obstacle was hospitalists lacking time and facing difficulties in reaching the PCPs. However, physicians showed desire to communicate directly by telephone at the time of discharge.

**Trial registration:**

French C.N.I.L. registration number 2108852. Registration date October 12, 2017.

## Introduction

The hospital discharge is a risky moment in the healthcare process. Medical responsibility is transferred from the inpatient provider or hospitalist to the primary care physician (PCP) [1]. In France, the PCP, also known as the treating physician or family physician, practices general medicine and occupies the central role of care coordinator. Poor coordination between healthcare professionals leads to discontinuity of care. It can be responsible for adverse events such as medical errors or drugs events, avoidable hospital readmissions and even death [2, 3]. At present, discharge summaries are the main communication medium between the hospitalists and PCPs. The quality of discharge summaries is often insufficient. They are incomplete, do not follow standardized formats and the most relevant informations are not clearly highlighted. Moreover, these documents are received late [4, 5]. This one-way communication is considered insufficient by PCPs in France [6, 7] as in other countries [8–10]. Efforts are being made, in France as in other countries [11], to improve the transmission of information between inpatient and outpatient physicians. Since 2016, the French health authorities have made it mandatory to write a “liaison letter” following a standardized format that must be given to the patient and sent to the PCP on the day of discharge. However, the rate of delivery of this “liaison letter” to the patient on the day of discharge remains insufficient: 45% of French hospitals stays in 2018 [12]. Despite the efforts made, the transmission of information at discharge remains incomplete and delayed, and physicians are dissatisfied [13, 14].

Studies have shown a need for direct physician-to-physician communication at the time of discharge [8–10, 13, 15]. This need for two-way communication is an expectation of both PCPs [8–10, 13] and hospitalists [10, 13]. This exchange could be done by telephone, email, message in the Electronic Medical Record (EMR), text message or fax [9, 13, 16]. PCPs would prefer the use of the telephone [8]. A phone contact at the time of discharge could ensure, through a two-way and interactive exchange, rapid transmission of information, improve patient safety and promote interprofessional collaboration by including the PCPs in the discharge planning process. Direct communication between physicians during hospitalization is found at low rates in studies ranging from 23 to 36.7% [9, 16, 17]. These low rates would be explained by a lack of time, barriers to contact PCPs, and the lack of a standardized process [10, 13]. This study proposes to evaluate a standardized process of direct, two-way, verbal phone communication to improve care transition. Its objective is to study the feasibility and benefits of a phone call from hospitalist to PCP to plan discharge.

## Methods

### Study design and settings

This study was a prospective, mono-centric, cross-sectional observational study. It took place in a 28-bed- acute medicine unit (“unité de post-urgence médicale” UPUM) of the Grenoble-Alpes University Hospital (GAUH) counting 2100 acute medicine beds.

### Participants

All patients discharged from UPUM to home between 11/20/2017 and 2/20/2018 were eligible. Patients transferred to other units and patients who did not declare a PCP were excluded.

### Intervention

For each eligible patient, the hospitalist responsible for the patient’s care was instructed to contact the patient’s PCP by telephone within 72 h before discharge. For this purpose, the hospitalist had a maximum of 3 telephone calls attempts. These 3 calls attempts had to be made more than an hour apart, during working hours (8:30 a.m. to 12:00 p.m. and 2:00 p.m. to 7:00 p.m., Monday to Friday) and spread over 48 h. The intervention was considered successful if a phone exchange between the PCP and the hospitalist occurred. The intervention was considered to have failed if no phone contact could be made after 3 call attempts. The phone exchange had to contain the key elements of hospitalization: reason and duration of hospitalization, medical care, drug changes, social care, date of discharge, and all necessary information to provide the follow-up. At the end of the phone call, the hospitalist directly asked the PCP for his opinion on the benefit of the calls by means of 4 short closed questions with a binary response modality, that were asked verbally. The content of the telephone calls was not recorded.

### Data source

The hospitalist had to complete a standardized paper form for each patient included. This form was designed for this study. It had been previously tested for one month in the same unit to ensure its validity and the understanding of the questions by 4 physicians who did not participate to the study. The first part of the form collected the times and dates of the calls. The second part included the collection of PCPs opinion. The third part included the collection of hospitalists opinion. It was collected by means of 12 questions concerning the feasibility and interest of the telephone contact. The occurrence of a change in hospital care (medical, therapeutic or social) as a result of the telephone exchange was also reported. Patient and hospitalization characteristics were collected from the hospital EMR. The variables collected were: gender, age (in years), place of residence (home, residence for independent seniors, or nursing home), presence of social assistance at home, number of treatments ordered on the patient’s entry prescription, length of stay (difference between arrival at the emergency room and discharge from the service, in number of whole days), social care during hospitalization (interview with a social worker, reassessment of allowance, assistance plan or living place), evaluation of functional status 15 days before admission with the Katz Activities of Daily Living (ADL D-15, scale rating from 0 to 6 with half points allowed) and Lawton Instrumental Activities of Daily Living (IADL D-15, scale rating from 0 to 8 with full points only) scales. The functional level was collected at entry by well-trained nurses from the unit. The main diagnosis of the stay was coded using the chapter titles of the International Classification of Diseases, 10th revision (ICD-10). The occurrence of a readmission at the GAUH within 30 days of discharge was recorded retrospectively after the 30 day period. All these data points were entered into a single computerized and anonymous database using an automatic entry grid. The homogeneity and quality of the data were checked.

### Statistical methods

Categorical variables were described by numbers and percentages; quantitative variables by the mean and standard deviation (SD). Univariate comparisons were conducted using the following tests: Chi2, Fisher’s exact test and Student’s t test. These tests were performed with R4Web software. The significance threshold was < 0.05.

### Authorizations

A registration to the “Commission Nationale de l’Informatique et des Libertés” was made. Study ethics approval was obtained. The informed consent was obtained from all participating physician.

## Results

### Participants

**Fig. 1:**
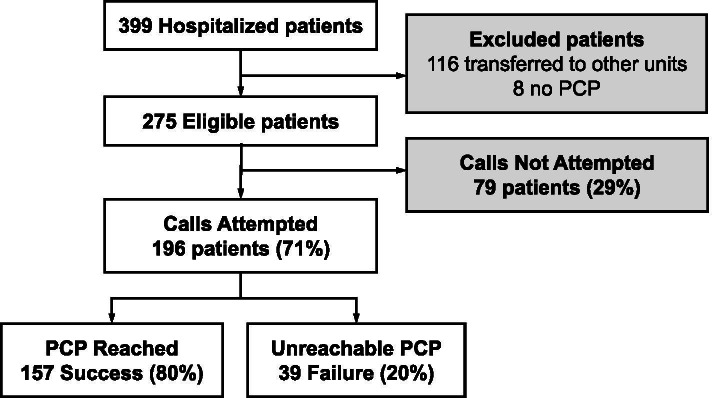
Flow Chart

During the study period, 275 of the 399 patients hospitalized in the unit were eligible (Fig. 1). 52% of eligible patients were male and 86% lived at home. Eligible patients had a mean age of 72.6 years and a mean functional status of ADL D-15 4.9. The average length of stay was 7.0 days and the number of 30-day readmissions was 53 or 19%.

### Intervention feasibility

#### Calls attempts

Call attempts were performed for 196 patients, representing 71% of eligible patients (Fig. 1). Call attempts resulted in successful contact with the PCP 157 times, representing 80% of call attempts and 57% of eligible patients. Two PCPs called the unit on their own initiative to check on their patient before discharge.

#### Telephone reachability of PCPs

The first call attempt was successful in 50% of cases, the second attempt in 48% of cases, and the third attempt in 31% of cases. The mean success rate for calls was 47% (total number of calls = 317). On average, the hospitalists had to complete 1.42 (± 0.63) calls to reach the PCPs. Calls had a better success rate from 3:00 to 5:00 pm and 6:00 to 7:00 pm. Calls had a better success rate on Wednesdays (58%) and Fridays (55%), but no significant difference between days were found (*p* = 0.344).

#### Respect of the intervention’s requirements

For 42% of call attempts (*n* = 81) some of the calls were completed after the patient was discharged because of a lack of response to the first call attempts. The other intervention requirements were properly fulfilled (92 to 98%).

#### Calls attempted versus not attempted: comparison of patient characteristics

Patients for whom calls were completed had longer hospital stays (7.5 days versus 6.1; *p* <  0.001), and were more often discharged on a workday than on a weekend (p <  0.001) (Table 1). There was no significant difference regarding other patient characteristics.

**Table 1:** Eligible patients’ characteristics and comparison

		Attempted calls patients (***n*** = 196)	Unattempted calls patients (***n*** = 79)	p
**Sex:** number of men’s (%)		103 (52%)	39 (49%)	0,730
**Age:** years, average (± D)		73,8 (± 17)	69,7 (± 21)	0,104
**Place of residence:** number (%)	Home	171 (87%)	65 (82%)	0,403
	Nursing home	16 (8%)	10 (13%)	
	Residence for independent seniors	9 (5%)	4 (5%)	
**Number of treatments ordered on entry prescription:** average (± SD)		6,6 (± 4)	6,0 (± 4)	0,281
**Presence of social assistance at home:** number of patients (%)		100 (51%)	44 (55%)	0,596
**ADL D-15:** average (± SD) ^a^		4,9 (± 1)	5,1 (± 1)	0,396
**IADL D-15**: average (± SD) ^b^		4,9 (± 3)	5,0 (± 3)	0,905
**Main diagnosis of the stay:** number (%)				0,409
	Diseases of the respiratory system	77 (39,3%)	33 (41,8%)	
	Diseases of the circulatory system	23 (11,8%)	9 (11,5%)	
	Diseases of the genitourinary system	16 (8,2%)	13 (16,4%)	
	Diseases of the digestive system	19 (9,7%)	6 (7,6%)	
	Infectious diseases	10 (5,1%)	2 (2,5%)	
	Diseases of the nervous system	9 (4,6%)	1 (1,2%)	
	Other diseases	42 (21,3%)	15 (19,0%)	
**Social care during hospitalization:** number (%)		41 (21%)	13 (16%)	0,499
**Length of stay:** whole days, average (± SD)		7,5 (± 4,0)	6,1 (± 3,9)	**0,008**
**Day on discharge:** number (%)				**< 0,001 **
	Workdays	183 (93%)	57 (72%)	
	Saturday	9 (5%)	18 (23%)	
	Sunday	4 (2%)	4 (5%)	
**Hospital readmission:** within 30 days, number (%)		36 (18%)	16 (20,2%)	0.735

^a^ missing data = 8 ^b^ missing data = 26

### Physicians’ opinions

#### Hospitalists’ opinions

The calls were conducted by 8 different hospital physicians. The average number of call attempts per hospitalist was 39.6. The opinion of the hospital doctors on the feasibility of the intervention was good (Table 2). They did not encounter difficulties to reach the PCPs, the additional workload was felt to be low with an estimated time of less than 10 min to complete the calls. The response to calls was described as good and cordial for all calls. The telephone exchange was perceived as useful, satisfying and providing security. It often resulted in the collection of new information, considered essential or useful by the hospitalists.

**Table 2:** Physicians’ opinions

HOSPITALISTS’ OPINIONS		Answer: Yes
***Opinion on the feasibility of the calls (n = 196)***		
The workload to successfully reach the PCP is low		85% (168)
The hospitalist did not encounter difficulties to reach the PCP		63% (123)
The time required to establish contact with the PCP is less than 10 min		63% (124)
***Opinion on the benefits of the calls (n = 157)***		
The call was useful and improved patient management		88% (138)
The hospitalist is satisfied with the call		97% (153)
The call is considered redundant with the “liaison letter” and the discharge summary.		53% (83)
The transmitted informations will be taken into account by the PCP in his management		94% (147)
The new informations collected during this call are useful or essential		57% (89)
The call gave the feeling of securing the patient’s discharge to the hospitalist		
	- in terms of medical care (n = 157)	91% (142)
	- in terms of medications (*n* = 146)	73% (106)
	- in terms of social care (*n* = 71)	50% (35)
**PCPs’ OPINIONS**		
Are you satisfied with this call?		100% (130)
As a result of this call, do you plan to see your patient again within 15 days? ^a^		78% (101)
Would you like this call to become systematic to prepare your patient discharge?		83% (109)
Would you prefer to receive this information in another way?		49% (64)
If yes, which one? ^b^	by email	51% (31)
	by web-based platform (« Zepra »)	39% (24)
	by postal mail	7% (4)
	by « liaison letter »	3% (2)

^a^ missing data = 4 ^b^ missing data = 3

#### PCPs’ opinions

132 different PCPs were reached. The PCPs contacted were mainly men (66%), working in urban or suburban areas (92%). The 130 PCPs who agreed to give their opinion were satisfied and would like to see these calls become systematic (Table 2). They preferred the use of the telephone as a first choice and email as a second choice. They thought they would see their patients again shortly after discharge thanks to the calls.

### Other points of interest

#### Hospital care changes

The telephone exchanges resulted́ in changes in patient care by hospitalist for 34 patients, or 22% of the telephone contacts. These changes were medical in 87% of cases (including changes in treatment) and social in 13% of cases (reassessment of allowance, assistance plan or living place).

#### Hospital readmissions

There was no statistically significant difference in re-admission to GAUH between the group of patients where the PCP was contacted (*n* = 157) and the group where the PCP was not contacted (calls not completed or failed, i.e. *n* = 118) within 30 days (*p* = 0.49). There also was no significant difference when only the subgroup of people over 75 years of age was included.

## Discussion

### Main results

This study shows that it is difficult to establish direct telephone communication between hospitalists and PCPs to plan discharge. Hospitalists were not able to complete the calls for all eligible patients. Calls were most often completed for patients with long lengths of stay and discharged during weekdays. These are situations where the hospitalists had more time to do them. As stated by Jones et al. [10], the main obstacle to their completion was the lack of availability of hospitalists. On the other hand, PCPs were not always reachable by phone, even when calling them repeatedly. More than half of the attempted calls were not answered. Mussman et al. encountered the same type of difficulties [18]. This poor rate of reachability can possibly be explained by part time practices or the absence of a regulation secretariat. To improve the availability of physicians of both sides, we could imagine encouraging physicians to reserve dedicated time slots for telephone exchanges between caregivers. Another possibility would be to arrange a telephone appointment before discharge by the secretariats or by sending an email to the PCP. Despite these barriers to communicate, when contact could be established, hospitalists and PCPs declared themselves satisfied. They showed a mutual interest in verbal communication at discharge. The PCPs surveyed would even like to see this exchange becoming systematic.

### Which patients to focus on?

The poor feasibility of the calls justifies the identification of a priority patient profile. According to Munchoff et al. physicians would like a direct communication at discharge for patients who are socially vulnerable and for whom hospitalists have a “concern” based on their clinical judgment [13]. According to Sheu et al., PCPs would like direct exchange for complex hospitalizations (multiple readmissions, multiple comorbidities, high-risk medication changes) [9]. Targeting patients at high risk of readmission and adverse events for whom continuity of care is a priority, such as the geriatric population, is warranted [19, 20].

### When to establish contact?

In our study, the changes in the hospital care recorded are an objective evidence of collaboration between PCPs and hospitalists. They are only possible if contact is established before discharge. In our study, as for Zackhof et al., contacts made before discharge allow better level of collaboration [21]. Discharge summaries are infrequently available at the first post-hospitalization appointment [4, 14], leading to an increased risk of readmission [14, 22]. Early contact should reduce the delay in transmission of information and improve patient safety. Moreover, calls prior to discharge could also allow physicians to clarify the dispatch and urgency of the follow-up [13]. This was the case in our study, as the PCPs reported seeing their patients promptly after discharge as a result of these calls.

### Hospital readmissions

The 30-day readmission rate reflects the local healthcare organization (coordination of caregivers, access to care, cooperation between primary and secondary caregivers). In our study, readmission rates were higher than national (15.8%) and local (15.6% for Isère) rates. These results may be explained by the high mean age of the patients of this study. Telephone contact did not reduce the readmission rate. It is possible that the study was underpowered. The intervention did not only target patients with high risk of readmission. By targeting this vulnerable population, the intervention might be more effective in reducing readmission rates. It has also been shown that the implementation of isolated interventions is not effective on readmission rates [23]. Multimodal and composite interventions are preferable, combining for example pre-discharge and post-discharge interventions [24]. Moreover, the proportion of avoidable readmissions is estimated to be only one quarter of all readmissions [25]. It was therefore unlikely that an isolated pre-discharge intervention could significantly reduce the readmission rate.

### Limitations

The main limitation of this study is that it took place in a single unit of a single center. It is uncertain whether the observations are transferable to other settings. The study probably suffered from a lack of power because the numbers of patients and physicians were modest. Readmissions were recorded only at the GAUH and not in other hospitals. Repetition of the surveys by a small number of hospitalists probably biased the responses by reducing their diversity. PCPs opinions were collected verbally through close-ended questions, which may have led to higher satisfaction rates. The daily workload in the unit, which likely influences calls completion, was not evaluated.

### How to communicate directly at patient discharge?

Sending discharge summaries only allows one-way communication from hospital to primary care providers. Such communication gives the information provider the status of “expert and holder of the truth”. A two-way information exchange would improve collaboration [26], by giving PCPs the opportunity to ask questions, exchange information and collaboratively plan follow-up [1, 21]. As in our study, Pantilat et al. reported that treating physicians preferred the use of the telephone [8]. Telephone numbers are easily accessible. Spoken language is interactive, spontaneous, transitory and allows for greater levels of collaboration [27]. Vocal intonation allows the transmission of subtle emotive cues that aid comprehension [28]. The content of a spoken communication differs from a written communication. This is well established in the field of nursing handovers. Verbal language allows us to communicate information that we would not be comfortable to write down [29], such as difficulties in management [30] or the psychological state of the patient [31]. It provides a more complete picture of the patient’s condition [29]. In hospital teams, it is considered the best way to transmit lots of information in a short amount of time [32]. It would further better compliance to recommendations [33]. Nevertheless, telephone communication has certain limitations. Telephone communication can be a source of breach in medical confidentiality [34]. The telephone is a frequent source of interruption of general practice consultations [35] and can affect the doctor-patient relationship [36] or be a source of errors [37]. Unlike written documents, the traceability of telephone exchanges is poor. Their private and unrecorded content is not accessible to patients, which can limit transparency and autonomy.

According to Munchhof et al. the preferred communication medium for PCPs would be email [13]. Written language has a better traceability and medico-legal value. Being asynchronous, it does not generate work interruptions. However, emails reduce interactions. Emails are not widely used for communication between healthcare providers [38]. The barriers to email use would be issues related to data security and confidentiality [38]. Another barrier is the lack of email directories for physicians. It is important to develop interface platforms for bi-directional and secure exchanges between primary and secondary care providers. In France, since 2014, a secure health messaging system (“Messagerie Sécurisée Santé”) is being deployed. It is a secure, national and free messaging system, reserved for healthcare professionals that incorporates a common directory. It is a promising tool that should promote interprofessional communication.

## Conclusions

The success of these calls depends on the availability of physicians from both sides which, according to our study, appears to be insufficient. This study showed that physicians desire to communicate directly by telephone at the time of discharge, despite difficulties in reaching out. These calls, which complement and highlight the contents of discharge summaries, should be conducted in priority for patients with complex hospitalizations or high risk of readmission. These calls, that are easily implementable in any discharge process, should be encouraged and become part of physicians’ routine practice to ensure effective transitional care.

## Data Availability

The datasets analyzed during the current study are available from the corresponding author on reasonable request.

## Abbreviations

ADL D-15: Katz Activities of Daily Living scale 15 days before admission
EMR: Electronic Medical Record
GAUH: Grenoble-Alpes University Hospital
IADL D-15: Lawton Instrumental Activities of Daily Living scale 15 days before admission
ICD-10: International Classification of Diseases, 10th revision
PCP: Primary care physician
UPUM: “Unité de Post-Urgence Médicale” acute medicine unit
SD: Standard deviation
